# Surgical treatment of diffuse idiopathic skeletal hyperostosis of cervical spine with dysphagia – Case report

**DOI:** 10.1016/j.amsu.2020.07.009

**Published:** 2020-07-14

**Authors:** Mikołaj Dąbrowski, Adam Sulewski, Jacek Kaczmarczyk, Łukasz Kubaszewski

**Affiliations:** a Department of Spine Orthopedics and Biomechanics, W. Dega University Hospital, Poznan University of Medical Sciences, 28 Czerwca 1956 135/147, 61-545, Poznan, Poland; b W. Dega University Hospital, Poznan University of Medical Sciences, 28 Czerwca 1956 135/147, 61-545, Poznan, Poland

**Keywords:** Cervical spine, DISH, Diffuse idiopathic skeletal hyperostosis, Forestier disease, Dysphagia, Case report

## Abstract

**Introduction:**

Diffuse idiopathic skeletal hyperostosis of cervical spine can lead to dysphagia.

**Presentation of case:**

A 73-year-old male weighing 110 kg and diagnosed with diffuse idiopathic skeletal hyperostosis in cervical spine with dysphagia. Patient manifested local pain of neck, a gradual limitation of spinal mobility. The surgery decision was based on swallowing problems, not pain in the spine. Before surgery radiographs, magnetic resonance images, computed tomography of the cervical spine and gastroscopy were obtained. Osteophytes were removed from the anterior approach with present otolaryngologist by surgery.

**Discussion:**

In this case used gastroscopy, CT and MRI for diagnostics. During the procedure we had support otolaryngologist. The patient has not been found a stenosis spinal canal and neurological symptoms. We were removed the ostheophytes. Interbody implants have not been applied.

**Conclusion:**

Disc degeneration disease itself can be asymptomatic or not a dominant problem for the DISH patients. Clinical signs may pharyngoesophageal and tracheal compression, causing dysphagia, shortness of breath and stridor. In this case, the cervical spine was stability and not demonstrated a stenosis in the spinal canal. Isolate removing of the osteophytes without implants in DISH of cervical spine can be enough solution.

## Highlights

• DISH of cervical spine may pharyngoesophageal and tracheal compression, causing dysphagia, shortness of breath and stridor.
• In this case, the cervical spine was stability and not demonstrated a stenosis in the spinal canal.
• Isolate removing of the osteophytes without implants in DISH of cervical spine can be enough solution.

## 1 Introduction

Diffuse idiopathic skeletal hyperostosis (DISH, Forestier disease) is a condition characterized by calcification and ossification of ligaments and enthuses affecting the vertebral column [1].

DISH is more frequent in men, affected 12–28% of adult population, and the incidence increases with age mainly affecting patients over the age of 60 years [2].

The exact cause is unknown. The mechanical, dietetic and long-term use of some antidepressants may be associated with the occurrence of the disease [3]. Isolated and predominant cervical spinal involvement may occur [4].

The clinical manifestations of DISH are variable [3]. Some patients are completely asymptomatic, while others complain of pain and stiffness. Pharyngoesophageal and tracheal compression may result in dysphagia, dyspnea, and stridor [5].

The incidence of dysphagia in patients with DISH is 0.2–28% [6]. Dysphagia in patients with DISH develops because of mechanical compression causing varying degrees of esophageal obstruction, impaired epiglottic motility, and distortion of the laryngeal cartilages [7]. Alterations of the upper cervical spine (C3–C4), may interfere with laryngeal function, while more distal changes (C5–C6) may induce spasm of the upper esophageal sphincter or esophageal compression [4].

Conventional radiography is usually sufficient to confirm the diagnosis of DISH [8]. A dynamic video fluoroscopy should be reserved for demonstrating the exact relationship of the cervical spine alterations with the swallow function in those patients who are experiencing dysphagia [8]. CT and MRI may be used to detect spinal canal stenosis and compressive myelomalacia [9].

Treatment for DISH is based on symptomatic relief of symptoms. There have been no well-designed studies evaluating the effectiveness of any therapy in this disease. Anterior cervical resection of osteophyte has a role in patients with airway obstruction and/or dysphagia, in whom conservative approach has failed [10].

## 2 Presentation of case

In a 73-year-old man with a body weight of 110 kg, DISH was diagnosed in the cervical spine. He had mild dysphagia for several years and during the six months before admission, the dysphagia had worsened. There was no history of dyspnea or stridor. The patient had hypertension arterialis.

In the cervical spine were severely limited range of motion, but movement of the cervical spine is rather painless. Root symptoms and motor or sensory deficiency signs negative. Difficulty swallowing, pain during swallowing. Eating with small bites. The reason for the patient's decision to surgical treatment was difficulties in swallowing, not pain in the spine.

In the medical history, the following co-existing conditions were found diabetes, arterial hypertension, paroxysmal atrial fibrillation. The patient underwent hemiarthroplasty of the left knee in 2010.

Radiographs, magnetic resonance images (MRI), computed tomography (CT) of the cervical spine and gastroscopy were obtained.

The lateral radiograph of the cervical spine revealed normal bone density and presence of new bone formation at C2-3, C3-4, C4-5 and C5-T1 vertebrae with anterior fusion. There were large shaped osteophytes protruding anteriorly and impinging upon the posterior wall of pharynx at C3-4 and C4-5 level causing narrowing at the level of osteophytes.

**Fig. 1:**
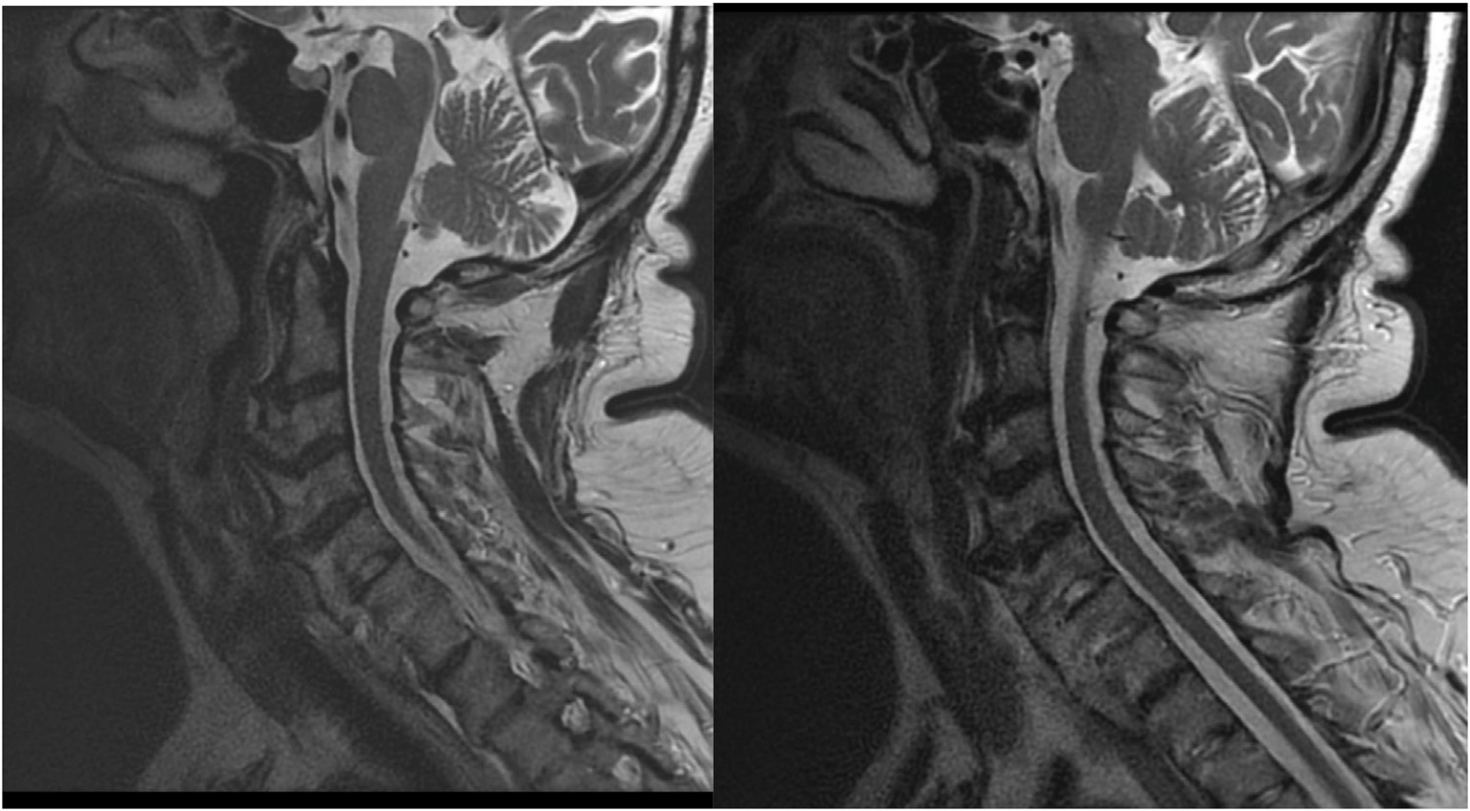
MRI scans of a 73-year-old man with diffuse idiopathic skeletal hyperostosis in cervical spine and dysphagia.

MRI of cervical spine examination was performed in sagittal and axial planes in T1 and T2 images - dependent and in sequence with fat saturation. Cervical lordosis preserved. The radiologist reported the massive osteophytes on the edges of the anterior and lateral C2–C7 vertebral bodies with the formation of bone bridges with the pressure on the esophagus and respiratory tract, smaller on the posterior edges with the features of bone and root conflict. Partial C6 and C7 ankylose. In T2-dependent images reduced signal level from intervertebral discs. Decreased height of C5/C6 intervertebral disc and to a greater extent C5/C6. No outbreaks of bone marrow edema. Correct image of the nerve roots in the area of the intervertebral foramina. No signs of intervertebral disc hernia. Without features of significant stenosis of the intervertebral foramina. The image of the spinal cord in the visible segment was correct (Fig. 1).

**Fig. 2:**
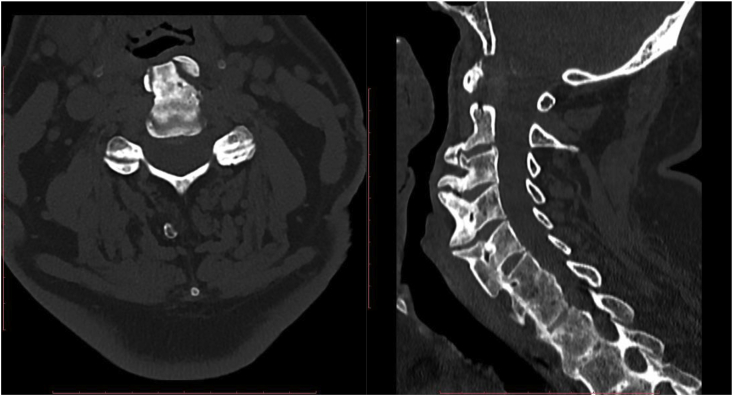
Sagittal computed tomography reconstruction of the cervical spine of a 73-year-old man with diffuse idiopathic skeletal hyperostosis and moderate dysphagia demonstrating extensive ossifications ventral of C2–C7.

CT showed large osteophytes forming bone bridges, on the front and lateral edges of the vertebral bodies C2 to C5, ossification of the anterior ligaments of the cervical spine at levels C5 to C7. The described changes oppress the esophagus and airways in the upper section. Multilevel deviations of the intervertebral space with a tendency to create bone blocks. Moderates osteophytes on the posterior edges of C6 and C7 vertebrae, grow into the spinal canal and little compress nerve elements. Multilevel degenerative changes in the intervertebral joints. At C3/C4 and C6/C7 level, a central bulge of the nucleus pulposus to the spinal canal with pressure on the tire sack is visible (Fig. 2).

Gastroscopy showed esophagus features acanthosis, loose inlet, irregular line, without erosions.

Gastritis and inflammation of the duodenal bulb. Polyp of pyloric ulcer.

### 2.1 Surgery

**Fig. 3:**
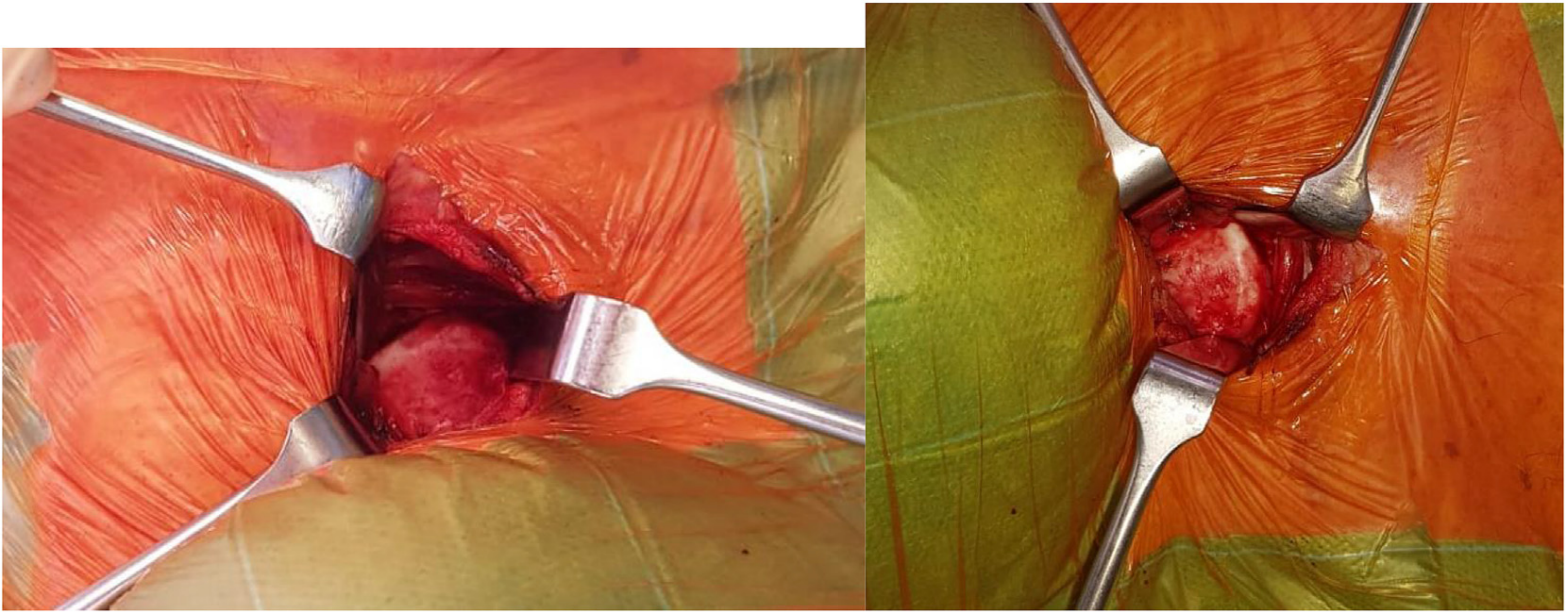
A view of C4/5 osteophyte during surgery of a 73-year-old man with diffuse idiopathic skeletal hyperostosis in cervical spine and dysphagia.

**Fig. 4:**
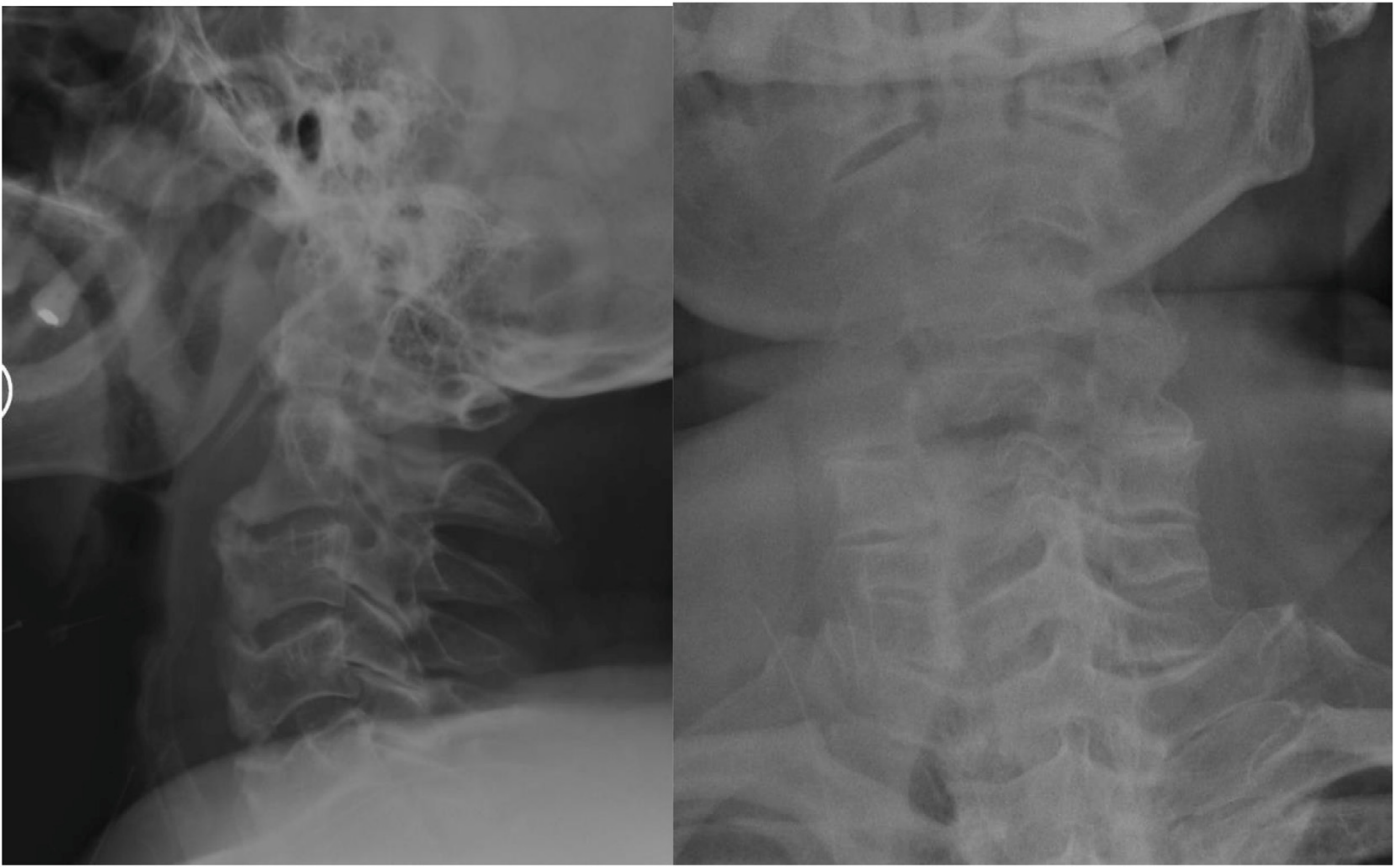
AP and lateral view X-ray of cervical spine after surgery of a 73-year-old man with diffuse idiopathic skeletal hyperostosis in cervical spine and dysphagia.

Surgical technique uses the standard Smith Robinson approach. We use a generous horizontal mid-cervical skin crease incision. Platysma is split longitudinally. This allows generous access from C2 down to C7-T1 (Fig. 3). We were able to achieve satisfactory exposure through a horizontal incision. By elevating the longus colli, the lateral aspects of the osteophytes are exposed. There were no adhesions with the esophagus, there were no problems with the surgical approach. To remove osteophytes, use a high-speed drill (Stryker). We used a drill to create a trough lateral to the osteophytes, using intensifier as guidance to drill the relatively avascular osteophytes and not enter the vascular vertebral body or the disc space. Osteophytes (“bone beaks”) were removed, but the osteophytes were not removed until the level of the anterior wall of the vertebral body (Fig. 4). Standard suturing was used. Sterile dressing. The drain was not used. The procedure was performed by 2 orthopedic surgeons having 8–10 years of experience in spine surgery. At the time of surgery was present otolaryngologist.

The day after the operation, the patient reported a significant improvement in swallowing and reduction of local pain within the cervical spine.

### 2.2 Quality of life (QoL)

A performance evaluation scale was used questionnaires Core Outcome Measures Index for the neck (COMI NECK) and (Neck Disability Index) NDI. Preoperative assessment was 7.3 in COMI NECK and 70 in NDI. 6 months after surgery Comi Neck was 1.4 and NDI - 10. The patient reported a significant improvement in swallowing, breathing and range of motion of cervical spine.

This case report was reported in line with the SCARE 2018 Checklist [11].

## 3 Discussion

In our case, we used gastroscopy for diagnostics. Some authors extend the diagnostics by videofluoroscopic esophagrams (VFEs) with barium swallom [12,13]. Gupta et al. justified, that barium swallow revealed smooth extrinsic indentation in the esophagus at the level of osteophytes. The difficulty in swallowing was attributed to the compression of the esophagus by the cricoid and cervical osteophytes [14]. We believe that such diagnostics may be important in the presence of short circuits at multiple levels and diagnostic difficulties, in which place the largest esophageal stenosis occurs.

In our case, we used CT and MRI. Butler et al. recommend routine preoperative imaging of the cervical spine in patients with a diagnosis of DISH to stratify risk of dysphagia [15].

Both diagnostic methods broaden knowledge. On the one hand, degenerative changes and potentially co-occurring changes in intervertebral discs and CT allowed the assessment of esophageal compression at major levels similar to the article by Weglowski and Piech [16].

We used anterolateral cervical approach as other authors [[15], [16], [17]].

At the time of surgery was present otolaryngologist in order to secure the potential complications, especially fears about adhesions with the esophagus. Chronic compression may result in an inflammatory process in the esophageal wall that can lead to fibrosis and adhesions, with fixation of the esophagus and disruption of the neural plexus [6]. Other authors did not describe the support of surgeons in other specialties.

We were removed the ostheophytes with not destabilizing the spine. Interbody implants have not been applied in our case because the patient has not been found a stenosis spinal canal and neurological symptoms. Intraoperatively stability and bone union simultaneously correct curvature of sagittal within the cervical spine.

With several osteophytes, consider whether to remove all osteophytes or those most susceptible to compression/stenosis based on CT or FEVS.

Like other authors, we have been wondering about the factors of recurrence of osteophytes [18].

Egerter, Kim et al. applicate of a thin layer of bone wax over the osteophytectomy sites may impede osteophyte formation [19]. Routine additional stabilization has been discussed as recurrence prevention. Prophylaxis using indomethacin or radiation, known primarily from hip replacement, also appears to be an option [20]. In our case, we did not use bone wax. In our opinion it has no effect on blocking bone growth.

## 4 Conclusion

DISH of cervical spine can lead to dysphagia. Disc degeneration disease itself can be asymptomatic or not a dominant problem for this patient.

In this case, instability of the cervical spine was not demonstrated, therefore removal of osteophytes without implantation was sufficient.

## Ethics

Written informed consent was obtained from the patient for publication of this case report and accompanying images. A copy of the written consent is available for review by the Editor-in-Chief of this journal on request.

## Authors contribution

AS MD surgery, MD AS data collection, MD AS concept, MD writing the paper, JK, ŁK redaction of article.

## Role of the funding source

The funding source had no involvement.

## Guarantor

Mikolaj Dabrowski.

## Provenance and peer review

Not commissioned, externally peer reviewed.

## Declaration of competing interest

Declarations of interest: none. The authors have no competing interests to declare.
